# Correlations of Expression Levels of a Panel of Genes (*IRF5*, *STAT4*, *TNFSF4*, *MECP2*, and *TLR7*) and Cytokine Levels (IL-2, IL-6, IL-10, IL-12, IFN-*γ*, and TNF-*α*) with Systemic Lupus Erythematosus Outcomes in Jordanian Patients

**DOI:** 10.1155/2019/1703842

**Published:** 2019-11-29

**Authors:** Amal H. Uzrail, Areej M. Assaf, Shtaywy S. Abdalla

**Affiliations:** ^1^Department of Biological Sciences, School of Science, The University of Jordan, Amman, Jordan; ^2^Department of Biopharmaceutics and Clinical Pharmacy, School of Pharmacy, The University of Jordan, Amman, Jordan

## Abstract

Systemic lupus erythematosus (SLE) is characterized by systemic end-organ damage. We investigated the involvement of *IRF5*, *TLR-7*, *MECP2*, *STAT4*, and *TNFSF4* genes and TNF-*α*, IFN-*γ*, IL-2, IL-12, IL-6, and IL-10 cytokines in SLE pathogenesis and in organ damage in Jordanian patients. Blood was collected from 51 patients and 50 controls. Expression levels of SLE genes in PBMCs and cytokine levels were determined using RT-PCR and ELISA, respectively. Expression levels of all genes and levels of TNF-*α*, IL-12, IL-6, and IL-10 were higher in SLE patients than those in controls (*p* < 0.05), whereas IL-2 level was lower. High *STAT4* (*α*), *TNFSF4*, and IL-10 levels correlated with cardiovascular damage, and high *MECP2* (*α*) and TNF-*α* correlated with renal damage. Pulmonary and musculoskeletal damages correlated with high levels of *TNFSF4*. We concluded that *STAT4* and *TNFSF4* genes with TNF-*α* and IL-10 cytokines could be used as biomarkers to assess SLE activity and manage treatment.

## 1. Introduction

Systemic lupus erythematosus (SLE) is an autoimmune disorder that belongs to immune complex-mediated (type III) hypersensitivity reactions. It is characterized by the deposition of immune complexes in different organs resulting in a broad range of clinical manifestations due to the loss of immunological tolerance and the presence of autoantigens, such as double-stranded DNA (dsDNA), chromatin-associated proteins, Ro (SSA), La (SSB), and Sm, and the RNA-associated proteins. Although the etiology of SLE is still not completely understood, multiple factors including genetics (e.g., *IRF5*, *TLR-7*, *MECP2*, *STAT4*, and *TNFSF4* genes), environmental, gender, and immunological factors such as cytokines and autoantibodies may play a role [1, 2]. It has been suggested that environmental factors may modulate the susceptibility to SLE disease through epigenetic changes (e.g., DNA methylation, histone modification, and micro-RNA-mediated regulation) [3].

Permanent organ damage may occur in SLE patients due to the disease itself or other pathologic processes such as atherosclerosis, hypercoagulability, hypertension, or even treatment. In addition, percentages and patterns of damage distribution vary according to ethnic, clinical, and sociodemographic factors [4]. Physicians use specific treatment protocols to treat SLE disease, which include corticosteroids to reduce inflammation quickly, as well as nonsteroidal anti-inflammatory drugs to reduce symptoms. The assessment of the degree of disease activity in a patient with SLE is important since the decision for the proper therapy depends on the accuracy of the physician's clinical judgment of disease activity [5].

On the other hand, a link between some genes and SLE in humans and mice has been established in several studies [1, 2]. Global profiling of gene expression in peripheral blood mononuclear cells (PBMCs) showed an upregulation in interferon- (IFN-) inducible genes in SLE patients compared to healthy controls [6, 7]. For example, Bennett and colleagues indicated that IFN expression is correlated with disease activity in pediatric lupus patients, and it was associated with more severe clinical manifestations in these patients [7], whereas a recent study uncovered a plasmablast signature as a robust biomarker for disease severity, which was reduced by the conventional therapies. This provides an opportunity for the development of personalized therapies by uncovering molecular networks that stratify lupus patients [8].

Numerous abnormalities in cytokine networks were found in patients suffering from SLE [9, 10]. For example, reviewing the role of IL-10 and TNF-*α*, Lόpez et al. [11] concluded that the conflicting data about the association of IL-10 and TNF-*α* genotypes with the observed clinical features support the heterogeneity of the disease and the involvement of diverse etiopathogenic factors. They also suggested that treatments and management of the disease might be individualized depending on IL-10 and TNF-*α* genotypes.

In a recent review, it was documented that T cells from SLE patients exhibit phenotypic and functional anomalies and that the disease itself affects the expression of genes and proteins and modifies the behavior of those cells [12]. Although the analysis of T cells from patients with SLE at a cellular and molecular level is challenging [12], the aim of this work is to examine the genetic basis of SLE by determining the expression levels of the genes *IRF5*, *TLR7*, *MECP2*, *STAT4*, and *TNFSF4* in SLE patients and to compare their expression levels to those from healthy individuals. There are few studies that examined the expression of SLE susceptibility genes (*IRF5*, *TLR7*, *MECP2*, *STAT4*, and *TNFSF4*) in lupus patients [13–16], and few studies are in the Arab countries about the involvement of cytokines in the pathogenesis of cytokines in SLE [17–19].

In Jordan, few studies dealt with the characteristics of disease in SLE patients, but the involvement of genes or cytokines in Jordanian SLE patients was not studied [20–22]. Therefore, it is warranted to shed some light on the role of some genes and cytokines that may contribute to the disease outcome in Jordanian SLE patients and to compare data with similar parameters for patients worldwide. Furthermore, the plasma levels of Th1 cytokines (TNF-*α*, IFN-*γ*, IL-2, and IL-12) and Th2 cytokines (IL-6 and IL-10) as well their associations with disease activity in SLE patients will be compared to those from healthy individuals. The data, albeit the relatively small number of patients, will be helpful in understanding the involvement of the tested genes and cytokines in the clinical manifestations and organ damage in SLE and will serve as a database for future follow-up studies. Furthermore, identification of biomarkers to predict onset, progression, and severity of disease are important for better management of this complex disease.

## 2. Materials and Methods

### 2.1. Study Design and Participants

The study was reviewed and approved by the Institutional Review Board of King Abdulla University Hospital (KAUH), Irbid, Jordan. SLE patients were recruited from the rheumatology clinic at KAUH between November 2014 and April 2015. Criteria for selecting participants for the study were as follows: all patients selected were adult (17 years old and above) and of Jordanian nationality who fulfilled the revised 1997 American College of Rheumatology (ACR) classification criteria [23]. The respondents for this study were 51 SLE patients. Age- and sex-matched healthy volunteers were recruited to serve as a control group (*n* = 50). The controls were healthy employees of the KAUH who had no history of disease, and their age and sex are described in Supplementary [Supplementary-material supplementary-material-1]. The disease activity was assessed in SLE patients by Systemic Lupus Erythematosus Disease Activity Index (SLEDAI) [24]. SLE patients were categorized into two groups based on the SLEDAI score [19]. Twenty-two (43%) SLE patients were with SLEDAI ≥ 10 and their mean age is 31.64 ± 2.44 years, served as high disease activity group. The remaining 29 (57%) SLE patients with a mean age of 35.86 ± 1.58 years were with SLEDAI < 10 and defined as low disease activity group.

### 2.2. Blood Collection

Venous blood (10–15 ml) was collected from SLE patients and healthy volunteers into heparinized vacutainer tubes (AFCO, Jordan). To determine cytokine levels, 3 ml of plasma was first separated and stored at −20°C until further analysis. The remaining blood was used within two hours for the isolation of peripheral blood mononuclear cells (PBMCs) as described below.

### 2.3. Isolation of PBMCs

Heparinized blood was diluted 1 : 1 with PBS (pH 7.2), and 15 ml of the diluted blood was layered over an equal volume of Ficoll-Hypaque gradient (Lymph prep; Accurate Chemical Corp., Westbury, NY). The gradient was centrifuged at 900 × *g* using a swing bucket centrifuge brakes off for 15 min at room temperature, and the buffy coat containing the peripheral blood mononuclear cells (PBMC) was collected and transferred into another centrifuge tube to be washed twice in 3 ml PBS each time by centrifugation at 400 × *g* for 10 min at 4°C with brakes on. Trypan blue exclusion was used to test for viability and count of the isolated cells. Twenty microliters of the washed cell suspension was added to an equal volume of 4% trypan blue stain (Sigma-Aldrich, USA) and loaded in a hemocytometer chamber (Marienfeld, Germany), and the viable cells were counted under an inverted light microscope (Olympus, Japan). The number of viable cells was 5 × 10^6^ cells/ml (viability >95%). All procedures were performed under aseptic environment in biological safety cabinet class I.

### 2.4. Semiquantitative RT-PCR for Gene Expression

Separated PBMCs were lysed, and total RNA was extracted using GenElute™ Mammalian Total RNA Miniprep Kit from Sigma-Aldrich (USA) according to the manufacturer's instructions. cDNA was prepared using reverse transcription kits (Promega, USA), according to the manufacturer's instructions. *IRF5*, *TLR7*, *MECP2*, *STAT4*, and *TNFSF4* mRNA expression levels were determined by semiquantitative RT-PCR using the IQ5 cycler (Bio-Rad, USA). SLE-related gene expression was normalized to *GAPDH* mRNA expression. The PCR was carried out using primers from Integrated DNA Technologies (IDT, USA) with sequences from the literature [1, 13, 14, 25, 26], as indicated in Supplementary [Supplementary-material supplementary-material-1]. Amplification of cDNA was carried out in the presence of SYBR green (KAPA Biosystems, USA). Each primer was run under the conditions indicated in Supplementary [Supplementary-material supplementary-material-1]. The results were expressed in arbitrary units (AU), based on the ratio of SLE gene mRNA: *GAPDH* mRNA for each gene. All procedures were performed under aseptic environment in biological safety cabinet class I.

### 2.5. Cytokine Determination

Cytokine levels were determined in 100 *μ*l of plasma from each participant per well for every cytokine, and all determinations were run in triplicate. The concentrations of human TNF-*α*, IFN-*γ*, IL-2, IL-12, IL-6, and IL-10 were determined using ELISA kits (eBioscience, San Diego, USA). In brief, ELISA plates were coated with 100 *μ*l/well of capture antibody, depending on the type of cytokine to be measured, and incubated overnight at 4°C. The wells' contents were then aspirated, washed three times with 250 *μ*l/well of wash buffer, and then blocked with 200 *μ*l/well of ELISA diluent. Plates were then incubated for one hour at room temperature before washing at least once with the wash buffer. Then, 100 *μ*l of each sample (patients and healthy controls) or standards (supplied with the kit) were added to the corresponding wells, incubated overnight at 4°C, and then washed three times. Detection antibody (100 *μ*l) diluted in ELISA diluent was added to each well, incubated for 1 hr at room temperature, and then washed again three times. Avidin horseradish peroxidase solution (100 *μ*l/well) diluted in ELISA diluent was added, incubated for 30 min at room temperature, and then washed five times before the addition of 100 *μ*l of the substrate 3,3′,5,5′-tetramethylbenzidine to each well for 15 min. Finally, 50 *μ*l of stop solution (2N H_2_SO_4_) was added and the plates were read using a microplate reader (Biotek, USA). The optical density at 450 nm, corrected by the reference wavelength of 570 nm, was measured. All cytokine assays were calibrated against the WHO international standards by the kit manufacturer. The sensitivity for the individual assays was 4 pg/mL for TNF-*α* and IFN-*γ* cytokines and 2 pg/ml for IL-2, IL-12, IL-6, and IL-10.

### 2.6. Statistical Analysis

Data were analyzed using SPSS statistical package (IBM, SPSS version 20, 2011). For numerical data, parametric data were expressed as means ± SEM, while nonparametric data were expressed as median and interquartile range. Qualitative data were given as frequency and percentage. Nonparametric numerical data were analyzed using the Mann–Whitney test. Correlation studies were performed using Spearman's rank correlation coefficient. *p* values less than 0.05 were considered statistically significant.

## 3. Results

### 3.1. Demographic and Clinical Characteristics of SLE Patients

Demographic data of patients and healthy controls are presented in Supplementary [Supplementary-material supplementary-material-1]. The mean age was 31.6 and 35.9 for patients with high and low disease activity, respectively, and the disease duration was 7–9 years. The different clinical manifestations and hematological and immunological features of the SLE patients who participated in this study are summarized in Supplementary [Supplementary-material supplementary-material-1]. Most patients suffered from arthropathy, malar rashes, ocular ulcers, renal problems as well as anemia.

### 3.2. Expression Levels of a Panel of Genes in PBMCs from SLE Patients and Healthy Controls by Semiquantitative RT-PCR

**Figure 1:**
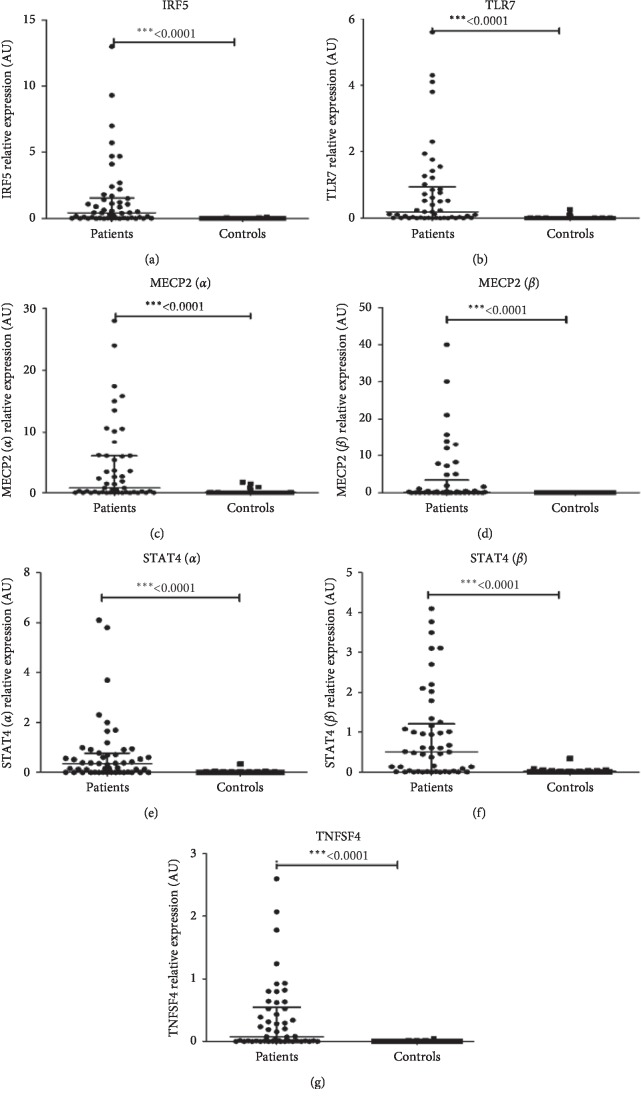
mRNA levels of SLE genes. mRNA expression levels were determined by semiquantitative RT-PCR using the IQ5 cycler: (a) *IRF5*, (b) *TLR7*, (c) *MECP2* (*α*), (d) *MECP2* (*β*), (e) *STAT4* (*α*), (f) *STAT4* (*β*), and (g) *TNFSF4* in PBMCs from SLE patients (*N* = 51) and healthy controls (*N* = 50) after normalization with *GAPDH* mRNA level. Data were analyzed by the Mann–Whitney test. Each symbol represents an individual sample, and horizontal lines indicate median values and 25 and 75 percentiles.

The expression level of the selected SLE genes was significantly higher in SLE patients compared to the healthy controls (Figure 1). No association between gene expression levels in SLE patients and SLEDAI was found.

### 3.3. Plasma Levels of Cytokines in SLE Patients and Healthy Controls

The levels of plasma cytokines in SLE patients compared to the healthy controls are shown in Table 1. Except for IFN-*γ* and IL-2, all cytokines were significantly increased in SLE patients compared to the healthy controls. There was a slight insignificant decrease in IFN-*γ*. The ratios of IL-10/IFN-*γ* and IL-10/IL-2 for healthy controls were 1.5 and 0.9, respectively, compared to 3.9 and 1.7 for SLE patients (*p*=0.0262, *p*=0.0007, respectively).

### 3.4. Cytokine Levels and Their Association with Disease Activity of SLE Patients

The differences in cytokine levels in SLE patients according to disease activity are shown in Table 2. Levels of TNF-*α* and IFN-*γ* were significantly lower, whereas IL-10 levels were significantly higher in patients with high disease activity than those with low disease activity. Also, the ratio of IL-10/IL-2 was significantly higher in SLE patients with high disease activity when compared to those with low disease activity.

### 3.5. Correlations between *IRF5, TLR7, MECP2* (*α*), *MECP2* (*β*), *STAT4* (*α*), *STAT4* (*β*), and *TNFSF4* Genes

All tested genes showed positive correlations with each other (Table 3). Furthermore, positively correlated pairs of tested genes showed significant linear correlations.

### 3.6. Correlations between TNF-*α*, IFN-*γ*, IL-2, IL-12, IL-6, and IL-10 Cytokines in SLE Patients

Table 4 shows that TNF-*α* was the only cytokine that showed positive correlations with three other cytokines: IL-2, IFN-*γ*, and IL-12. The other cytokines correlated with either one or maximally two cytokines.

### 3.7. Correlations between Studied Genes and Cytokines in SLE Patients

No correlation was found between cytokine levels and gene expression levels. The only exception was a negative correlation of IL-12 with *TLR7*, *MECP2* (*α*), *STAT4* (*α*), and *TNFSF4* (Table 5).

### 3.8. Correlations between Plasma Cytokine Levels and SLEDAI

A positive correlation was found between SLEDAI score and IL-10 (*R* = 0.339, *p*=0.015), and the ratio of IL-10/IFN-*γ* (*R* = 0.435, *p*=0.001). On the other hand, TNF-*α* and SLEDAI score were negatively correlated (*R* = -0.306, *p*=0.029). No significant correlations were found between plasma levels of IFN-*γ*, IL-2, and IL-12 and IL-10/IL-2 ratio with SLEDAI score.

### 3.9. Correlations between Gene Expression, Cytokine Levels in SLE Patients, and Their Serological biomarker Profile (ANA and Anti-dsDNA)

No significant correlation was found between the highly expressed levels of the studied genes, cytokine levels, and anti-dsDNA in SLE patients. However, there was a positive correlation between IL-6 and ANA (*R* = 0.298, *p*=0.036), and a negative correlation between the highly expressed *IRF5* and ANA (*R* = −0.327, *p*=0.022) (Supplementary [Supplementary-material supplementary-material-1]).

### 3.10. The Relationship between Gene Expression, Cytokine Levels, and Clinical Manifestations and Organ Damage Domains of SLE Patients

The associations between the expression levels of the studied genes, plasma levels of cytokines, and the clinical manifestations in SLE patients are shown in Table 6. Data show a significant correlation between hypothyroidism and expression levels of *IRF5*, *TLR7*, *MECP2* (*α*), *STAT4* (*α*), *STAT4* (*β*), and *TNFSF4* genes as well as with the cytokine IL-10. Malar rash correlated significantly with *IRF5* and *STAT4* (*α*). Ulcerations of fingers associated significantly with expression levels of *α*- and *β*-isomers of *STAT4*. Cardiovascular damage associated significantly with expression levels of *STAT4* (*α*) and *TNFSF4* genes and with IL-10 cytokine, whereas renal damage was associated with *MECP2* (*α*) gene and with TNF-*α* cytokine and pulmonary and musculoskeletal damage associated significantly with *TNFSF4.*

## 4. Discussion

Systemic lupus erythematosus is an autoimmune disease affecting multiple organs and systems [27]. The etiology of the disease is multifactorial with the involvement of genes and environmental factors. Furthermore, the immune malfunction is mediated by alterations in the production of some cytokines [7, 9, 11]. Therefore, a complicated disease index system is used to classify its severity. The disease has clinical heterogeneity, and many different pathways may lead to disease expression. This heterogeneity made it necessary to individualize treatment protocols, i.e., personalized medicine. There are international recommendations for a treat-to-target strategy in SLE, with a long-term goal of achieving remission. Although improvements occurred in treating SLE, the available conventional drugs do not control the disease completely in many patients, and the outcomes are not successful with off-target effects occasionally. This necessitates a better understanding of the biological bases of SLE which should be translated into more effective care for patients [27]. For this to be accomplished, this study investigated the demographics, the clinical manifestations, organ damage patterns, and the biomolecular data of patients.

### 4.1. Demographic Aspects

The majority of the patients in this study were females with a ratio of 16 : 1. The increased frequency of SLE among women has been indicated in several publications [28, 29]. The female gender-dependent bias in lupus depends not only on the X-chromosome but also on the wide range of effects of sex hormones, particularly estrogen, on the immune system and target organs [30]. The occurrence of three predisposing genes (*IRAK1*, *MECP2*, and *TLR7*) on X-chromosome is expected to have a role in the increased frequency of disease among females, thus raising the possibility of a gene dosage effect [31]. It has been found that sex hormones play a role in regulating the molecular mechanisms of the innate and adaptive immune systems, and control immune responses in health, but complex interactions of hormones and environmental factors in genetically predisposed individuals may cause deregulation in the immune responses, leading to immune-mediated diseases like autoimmune diseases [30]. The role of estrogen in SLE was suggested by a number of observations. Lahita, for example, showed that the female-to-male ratio of SLE patients differs according to the age group where the effect of estrogen was noticed. The ratio in adults, especially in women of child-bearing years, ranged from 7 : 1 to 15 : 1, but it was 3 : 1 in children. In “older” individuals, especially in postmenopausal women, the ratio was about 8 : 1 [32]. Estrogen plays an important role in the development and functions of B cell and contributes to their dysfunction in autoimmune diseases [30]. Using murine models, this sex bias has been attributed to the upregulation of BAFF expression by estrogen and interferon signaling through upregulation of p202 protein which is encoded for by *Ifi202* gene (IFN-regulated gene) [33]. In addition, estrogen influences T-cell development and functions as well as the immunomodulatory cytokine production which contributes to disease pathogenesis and organ pathology in lupus. In addition, polymorphism in the ER*α* (Esr) gene has been linked with SLE and been found to be associated significantly with the development of disease, age at disease onset, or disease features and severity [30]. Therefore, blocking estrogen receptors in a targeted manner may yield better therapeutic treatments.

In addition, the mean age was 31.6 and 35.9 for patients with high and low disease activity, respectively, and the disease duration was 7–9 years. The mean age of SLE patients with high disease activity was insignificantly less than those with low activity, in consistence with previous studies [19]. Age was found to be negatively correlated with disease severity (*R* = −0.336, *p*=0.016). Disease duration was relatively longer among high disease activity group than those with low disease activity, in consistence with the findings of a recent study [34].

Most patients suffered arthropathy, malar rashes, ocular ulcers, and renal problems as well as anemia. Studies indicated that frequencies and patterns of distribution of organ damage among SLE patients vary according to ethnic, clinical, and sociodemographic factors [35, 36]. Although SLE was the subject of many studies on Arab ethnicity [7–9, 20–22], the present study examines the prevalence of organ damage among SLE patients besides examining the roles of certain genes and cytokines in contributing to the disease outcomes and their correlations with clinical manifestations. In this study, patients were categorized according to their organ damages and classified into domains. Each patient was found to suffer from one organ damage or more. So, skin was extensively affected in the majority of cases (skin ulceration was 33.3%) followed by renal domain showing proteinuria, in consistence with a study from Brazil [35]. Skin damage may be due to overexposure to UV light especially UVB, which was indicated to alter the chemistry of DNA as well as Ro and nRNP antigens, enhancing their immunogenicity [37]. The patients showed different patterns of clinical manifestations and organ damage. This might be due to different genetics, age, time to initiate treatment, use of hydroxychloroquinone, type of induction therapies used, extent of organ involvement, adherence to medication, and psychosocial factors affecting disease control [27].

### 4.2. The Role of Genes

It is proposed that diseases of complex origin such as SLE have a component of quantitative genetics that is responsible for their susceptibility and variation in their phenotype. Although all allelic forms responsible for the variability of a particular complex phenotype were identified, they could not explain all the phenotypic variance by an additive effect [38]. Studies have indicated the contribution of many different genes to the risk of inheriting SLE although the size effect of these individual genes may be small [39]. Monolio et al. found that the 6 most strongly associated genes can only explain about 15% of the heritability of SLE [40]. Nevertheless, the contribution of SLE genes is considered one of the most important factors in disease susceptibility by virtue of their involvement in the production of autoantibodies and immune complexes, thus initiating the disease process [31]. Furthermore, it has been suggested that understanding the genetic origin of SLE is pivotal to develop new biological therapeutic approaches directed against molecular mediators of the disease since the conventional treatments are associated with side effects [41]. SLE patients, in the present study, demonstrated a significant increase in the expression of SLE susceptibility genes *IRF5*, *TLR7*, *MECP2*, *STAT4*, and *TNFSF4* when compared to healthy volunteers, with *MECP2* (*α*) expression having the highest value followed by *IRF5* and *MECP2* (*β*). *STAT4-β* isoform expression was almost two-fold that of *STAT4-α* isoform. These observations are consistent with other studies, thereby stressing the contribution of the same genes to SLE pathogenesis [13–15, 25, 42, 43]. *IRF5* contribution to SLE disease can be explained by the fact that its protein product functions as a transcriptional factor which activates cytokines such as IL-6, IL-12, and TNF-*α* via the activation of Toll-like receptor signaling pathway [16]. These cytokines, which increased significantly in the patients in this study (Table 1), play a central role in the initiation and progression of SLE disease [44]. On the other hand, high level of *STAT4* leads to the production of high levels of IFN-*γ* that promotes Th1 and Th17 cell expansion, thus contributing to autoimmune disease [45]. In the present study, such an increase in IFN-*γ* was not observed, possibly due to the small effect size of this individual gene [38]. Recent reports have confirmed the additive effects of *STAT4* and *IRF5* SNPs which may increase the risk of SLE and antiphospholipid syndrome [14, 46]. Alternatively, the increased expression of *MECP2* gene may contribute to the impaired Th1 responses and reduced levels of IFN-*γ* [47]. Finally, increased levels of TNFSF4 might lead to increased costimulation of CD4^+^ T cells and further activation of antigen-presenting cells expressing TNFSF4 such as B cells, and inhibiting T regulatory cells which destabilizes peripheral tolerance [31].

SLE disease is a complex polygenic disease that may be caused by multiple gene-gene interactions, where each single gene has only a small contribution to the pathogenesis of the disease. Several studies identified the expression of individual susceptibility genes in SLE patients [25, 26, 42, 47]. On the other hand, few studies identified the combined effect of some susceptibility genes on SLE disease, where they tested the combined effect of genes other than those tested in the present work [46, 48]. In the present study, the influence of SLE genes (*IRF5*, *TLR7*, *MECP2* (*α*), *MECP2* (*β*), *STAT4* (*α*), *STAT4* (*β*), and *TNFSF4*) on each other in developing SLE disease was examined. A positive correlation was found between all pairs of the studied genes, suggesting that the expression of one gene may influence the expression of another. For example, a significant positive linear association between *IRF5* and both isomers of *STAT4* has been shown here (Table 3) which confirms the additive effects of both genes, as previously reported [14]. Since similar correlations were observed between gene pairs, it is likely that the mere expression of one susceptibility gene would not be enough to induce the disease process in SLE patient. In addition, it is suggested that epigenetic changes may contribute to variation in gene expression between individuals and to complex phenotype variability and its heritability [38]. Therefore, and as concluded by others, understanding the genetic profile of individual patients may allow the development of more targeted and personalized approaches to treatment [39].

### 4.3. The Role of Cytokines

SLE disease develops as a result of the imbalance of Th1 and Th2 cytokines [48]. In this study, both Th1 cytokines (TNF-*α* and IL-12), and Th2 cytokines (IL-6 and IL-10) were significantly higher in the plasma of SLE patients than healthy volunteers, with a sharper increase in Th2 cytokines, confirming a shift in the Th2/Th1 balance toward Th2 cytokines (Table 1). This observation is consistent with studies suggesting that the population size of Th1 cells in SLE patients is reduced whereas the effector function of Th2 cells is enhanced in SLE patients [49]. However, a recent report demonstrated a decrease in IL-10-producing B cells in lupus nephritis patients compared to healthy controls, reflecting an impaired regulatory function of those cells too [50]. Furthermore, a recent study showed that IL-8, IP-10, MIG, MIP-1*α*, and RANTE levels were significantly correlated with SLE activity; their concentrations in SLE patients with low and moderate/high activity differed significantly [51]. This is consistent with the observation, in the current study, that the correlations between disease activity and cytokine levels showed a significant reduction in TNF-*α* and IFN-*γ* levels, whereas IL-10 levels were significantly higher in patients with high disease activity than those with low disease activity. Also, the ratio of IL-10/IL-2 was significantly higher in SLE patients with high disease activity when compared to those with low disease activity.

Since correlation studies showed only a positive correlation between the cytokines of the same subtypes of helper cells (Table 4), this indicates the interplay of those cytokines within the same T helper cell type. The significance of monitoring cytokines profile has been stressed, and it was suggested that cytokines may be used as markers for early detection of flares (active form of disease), which might distinguish between flares and chronic damage, as well as to monitor therapy [52].

### 4.4. Interaction of Genes and Cytokines

To further investigate the pathogenesis of SLE, interaction between cytokines and genes was studied (Table 5). With the exception of IL-12, which showed a negative correlation with *STAT4*, *TLR7*, *MECP2* (*α*), and *TNFSF4*, no statistical correlation was observed between most of the genes and cytokines. It has been found that the level of STAT4 protein, which is a critical IL-12 signaling component, dramatically decreased 24 hours after IL-12 stimulation, whereas levels of mRNA of *STAT4* were not affected. This decrease in *STAT4* protein might be due to specific degradation of phospho-STAT4, via the proteasome degradation pathway. Decreased level of *STAT4* protein leads to a decrease in STAT4 DNA binding activity and thus reduced proliferation and secretion of IFN-*γ*. This downregulation of *STAT4*, which is specific for IL-12 signaling, might be due to the prolonged activation of *STAT4* induced by IL-12 [53]. In support of this, it was interesting to notice that IFN-*γ* was slightly higher in healthy controls than in patients and significantly higher in patients with inactive disease than those with active disease, although *STAT4* was significantly higher in the patients in the present study.

In correlations between cytokine levels and gene expression levels with the most important laboratory biomarkers of SLE such as ANA and anti-dsDNA, we found no correlation for anti-dsDNA antibody titer. This is at variance with other studies that found the best correlation between anti-dsDNA antibody titers and SLEDAI in African-American patients who also responded better to B-cell depletion therapies than Caucasian patients [8]. This is also inconsistent with a study on Malaysian patients where they detected anti-dsDNA antibodies in less than one-third of lupus nephritis patients with higher frequency in patients with active disease [34]. On the other hand, ANA correlates positively with IL-6 level but negatively with *IRF5* gene expression. IL-6 is known as an important inflammatory cytokine that contributes to the inflammatory process of disease. In addition, it plays a central role in regulating both humoral and cellular-mediated immune responses such as B-cell activity and T-cell growth and differentiation. Since autoantibody production relies on B-cell differentiation and activation, therefore, high serum levels of IL-6 may account for the high levels of ANA in SLE patients [54]. The negative correlation between *IRF5* and ANA can be attributed to the role of *IRF5* as a susceptibility gene linking type I IFN pathway and disease pathogenesis in SLE patients [55].

The relation between the genes expressed and cytokine levels in SLE patients with the clinical manifestations showed significant association with at least one clinical manifestation except for *STAT4* (*α*) which was associated with four features: malar rash, alopecia, hypothyroidism, and finger ulceration when compared to those without such manifestations. Hypothyroidism was also significantly associated with high levels of gene expression of *IRF5*, *TLR7*, *MECP2* (*α*), *STAT4* (*β*), and *TNFSF4*. A recent study indicated that thyroid dysfunction is frequent in SLE patients [56]. This suggests that *TNFSF4* is the gene mostly associated with the most severe form of the disease in this study. There is an unmet need to explore diagnostic markers such as biomarkers that can predict the disease onset or to identify early stages of the disease and biomarkers of prognostic value, particularly those predicting flares or new onset of organ involvement. Developing biomarkers that identify pathogenetically subsets of patients in order to improve approaches to clinical trials through matching interventions with the appropriate immunological targets [27] seems to be an ultimate goal. In addition, to provide much more information about these different subsets, this requires expanding the measurements of candidate biomarkers of appropriate capabilities to a large-scale platform. These approaches can be accomplished using an array of gene expression, autoantibodies of different immunological classes, and soluble mediators like cytokines [27]. Detecting the levels of IFN-inducible genes such as *IRF5* and *STAT4* and cytokines such as TNF-*α* and IL-10 may be important toward the implementation of a more personalized therapeutic protocol that is safer than the conventional treatments. For example, it has been indicated that the use of short-term induction therapy with anti-TNF-*α* in SLE patients with severe joint involvement was a safe therapeutic approach [41], and biological treatments targeting type I IFN are currently in trial [27].

## 5. Conclusions

The present work demonstrated increased levels of expression of a panel of genes (*IRF5*, *TLR7*, *MECP2* (*α*), *MECP2* (*β*), *STAT4* (*α*), *STAT4* (*β*), and *TNFSF4*) in 51 SLE patients, but with no correlation between these levels and the severity of SLE disease. We also showed that Th1 cytokines, such as TNF-*α* and IL-12, and Th2 cytokines, such as IL-6 and IL-10, are also increased with a shift toward Th2 cytokines, indicating enhanced Th2 function in these patients. Correlation studies between pairs of genes and between cytokines and associations of genes and cytokines with clinical manifestations indicate that *STAT4* and *TNFSF4* genes and cytokines such as TNF-*α* and IL-10 might be helpful biomarkers to assess disease activity and manage treatment protocols. This is interesting since a recent study showed that populations from different geographic regions share common genetic risk factors for SLE [57]. Finally, this work may have diagnostic and therapeutic implications and provides some insights into the pathogenesis of SLE patients and gives some guidance to clinicians in determining the disease activity and development and in managing treatment protocols based on cytokines and gene expression profiles for patients.

## Figures and Tables

**Table 1:** Plasma levels of cytokines in SLE patients and healthy controls. Cytokine levels were determined in 100 *μ*l of plasma from each participant. The concentrations of human TNF-*α*, IFN-*γ*, IL-2, IL-12, IL-6, and IL-10 were determined using ELISA. *N* was 50 and 51 for the controls and the patients, respectively.

Cytokine type	Cytokine	Control	IQR (pg/ml)	SLE patients	IQR (pg/ml)	*p* ^*∗*^
		Median (pg/ml)		Median (pg/ml)		
Th1 cytokines	IL-2	16.90	(11.39, 26.01)	11.15	(2.76, 24.33)	**0.0374**
	IFN-*γ*	26.80	(13.64, 70.20)	25.39	(13.08, 44.33)	**0.4190**
	TNF-*α*	8.35	(1.99, 26.90)	9.31	(4.78, 32.56)	**0.0029**
	IL-12	0.00	(0.00, 3.34)	2.86	(0.00, 20.91	**0.0078**

Th2 cytokines	IL-6	2.84	(1.61, 4.80)	7.21	(4.54, 14.56)	**< 0.0001**
	IL-10	25.08	(9.00, 47.15)	43.03	(29.29, 59.06)	**0.0008**

^*∗*^ *p* < 0.05 is considered statistically significant using the Mann–Whitney test. IQR: interquartile range (25 percentile and 75 percentile).

**Table 2:** Cytokine levels (pg/ml) in SLE patients with low or high disease activity. Cytokine levels were determined in 100 *μ*l of plasma from each participant. The concentrations of human TNF-*α*, IFN-*γ*, IL-2, IL-12, IL-6, and IL-10 were determined using ELISA.

Cytokine		Low activity (*n* = 29)	High activity (*n* = 22)	*p* ^*∗*^
Th1	TNF-*α*	17.5 (6.79, 42.84)^*∗∗*^	6.5 (3.47, 11.93)	0.017
	IFN-*γ*	31.1 (15.81, 48.89)	19.1 (8.17, 33.57)	0.047
	IL-2	10.9 (2.05, 29.36)	11.2 (4.14, 17.62)	0.542
	IL-12	2.9 (0, 16.83)	8.2 (0, 32.37)	0.576

Th2	IL-6	7.2 (4.27, 14.77)	8.2 (4.65, 15.02)	0.621
	IL-10	39.8 (22.25, 49.22)	52.6 (34.66, 85.85)	0.023

Th2/Th1	IL10/IL-2	2.2 (0.79, 9.59)	4.7 (2.71, 14.9)	0.045

High disease activity = SLEDAI ≥ 10, low disease activity = SLEDAI < 10. ^*∗*^*p* < 0.05 is considered statistically significant using the Mann–Whitney test. ^*∗∗*^Values are median and (25 percentile and 75 percentile).

**Table 3:** Correlations between studied genes. Correlations were performed using Spearman's rank correlation coefficient.

Gene	R&P	*STAT* (*α*)	*STAT* (*β*)	*MECP2* (*α*)	*MECP2* (*β*)	*TNFSF4*	*TLR7*
*IRF5*	*R*	0.497^*∗∗*^	0.577^*∗∗*^	0.669^*∗∗*^	0.375^*∗∗*^	0.628^*∗∗*^	0.725^*∗∗*^
	*P*	0.000	0.000	0.000	0.007	0.000	0.000

*STAT* (*α*)	*R*		**0.676** ^*∗∗*^	**0.539** ^*∗∗*^	**0.424** ^*∗∗*^	**0.588** ^*∗∗*^	**0.558** ^*∗∗*^
	*P*		0.000	0.000	0.002	0.000	0.000

*STAT* (*β*)	*R*			**0.702** ^*∗∗*^	**0.339** ^*∗*^	**0.660** ^*∗∗*^	**0.687** ^*∗∗*^
	*P*			0.000	0.015	0.000	0.000

*MECP2* (*α*)	*R*				**0.466** ^*∗∗*^	**0.689** ^*∗∗*^	**0.789** ^*∗∗*^
	*P*				0.001	0.000	0.000

*MECP2* (*β*)	*R*					**0.411** ^*∗∗*^	**0.601** ^*∗∗*^
	*P*					0.003	0.000

*TNFSF4*	*R*						**0.741** ^*∗∗*^
	*P*						0.000

^*∗*^Correlation is significant at the 0.05 level (2-tailed). ^*∗∗*^Correlation is significant at the 0.01 level (2-tailed).

**Table 4:** Correlations between studied cytokines in SLE patients. Correlations were performed using Spearman's rank correlation coefficient.

Cytokines	R&P	IFN-*γ*	IL-2	IL-12	IL-6	IL-10
TNF-*α*	*R*	**0.601** ^*∗∗*^	**0.286** ^*∗*^	**0.419** ^*∗∗*^	0.014	−0.251
	*P*	**0.000**	**0.042**	**0.002**	0.924	0.075

IFN-*γ*	*R*		**0.189**	**0.455** ^*∗∗*^	0.056	−0.008
	*P*		0.185	0.001	0.696	0.956

IL-2	*R*			0.179	−0.092	−0.011
	*P*			0.208	0.521	0.940

IL-12	*R*				−0.020	0.102
	*P*				0.887	0.475

IL-6	*R*					**0.384** ^*∗∗*^
	*P*					0.005

^*∗*^Correlation is significant at the 0.05 level (2-tailed). ^*∗∗*^Correlation is significant at the 0.01 level (2-tailed).

**Table 5:** Correlations between expression levels of the studied genes and cytokines levels. Correlation studies were performed using Spearman's rank correlation coefficient after normalization with the housekeeping gene *GAPDH* in SLE patients.

Gene/Cytokine		*IRF5*	*TLR7*	*MECP2* (*α*)	*MECP2* (*β*)	*STAT* (*α*)	*STAT* (*β*)	*TNFSF4*
TNF-*α*	*R*	−0.272	−0.153	−0.079	0.048	−0.204	−0.058	−0.123
	*P*	0.054	0.285	0.581	0.736	0.151	0.685	0.391

IFN-*γ*	*R*	−0.061	−0.056	0.037	0.014	−0.235	−0.113	−0.072
	*P*	0.672	0.697	0.795	0.925	0.097	0.429	0.617

IL-2	*R*	0.034	0.040	−0.085	0.014	−0.075	0.084	−0.074
	*P*	0.815	0.781	0.552	0.925	0.601	0.556	0.607

IL-12	*R*	−0.239	**−0.365** ^*∗∗*^	**−0.281** ^*∗*^	0.018	**−0.331** ^*∗*^	−0.231	**−0.394** ^*∗∗*^
	*P*	0.091	**0.008**	**0.046**	0.902	**0.018**	0.103	**0.004**

IL-6	*R*	0.002	−0.085	−0.210	0.136	0.080	−0.028	0.052
	*P*	0.988	0.555	0.140	0.341	0.575	0.846	0.717

IL-10	*R*	0.146	0.112	0.133	0.115	0.109	0.127	0.088
	*P*	0.308	0.435	0.352	0.423	0.446	0.376	0.538

^*∗*^Correlation is significant at the 0.05 level (2-tailed). ^*∗∗*^Correlation is significant at the 0.01 level (2-tailed).

**Table 6:** Relation between clinical manifestations and organ damage domains of SLE patients and expression levels of SLE genes and levels of cytokines.

Gene	Clinical manifestation	*N*	*p* ^*∗*^	Organ damage	*N*	*p* ^*∗*^	Cytokine	Clinical manifestation	*N*	*p* ^*∗*^	Organ damage	*N*	*p* ^*∗*^
***IRF5***	Malar rash	23	0.023	—	—	—	**TNF-*α***	Nephropathy	22	0.026	Renal	16	0.013
	Hypothyroidism	8	0.003										

***TLR7***	Hypothyroidism	8	0.010	—	—	—	**IL-6**	Arthritis	13	0.013	Cardiovascular	7	0.041
								Arthralgia	24	0.025			

***MECP2* (*α*)**	Hypothyroidism	8	0.023	Renal	16	0.038		—	—	—	—	—	—

***STAT4* (*α*)**	Alopecia	11	0.048	Cardiovascular	7	0.007	**IL-10**	Hypothyroidism	8	0.004	—	—	—
	Malar rash	23	0.039										
	Hypothyroidism	8	0.015										
	Ulceration of fingers	7	0.003										

***STAT4* (*β*)**	Hypothyroidism	8	0.015	—									
	Ulceration of fingers	7	0.003										

*TNFSF4*	Hypothyroidism	8	0.010	Musculoskeletal	6	0.043							
				Cardiovascular	7	0.011							
				Pulmonary	6	0.037							

^*∗*^ *p* value < 0.05 is considered significant using the Mann–Whitney test. *N* is the number of patients showing the clinical manifestation or the organ damage.

## Data Availability

The data from all the patients and volunteers and the raw data used to support the findings of this study are available from the corresponding author upon request. In addition, 5 Supplementary Tables are appended with the manuscript.

## Supplementary Materials

Supplementary Materials Table 1: the demographic data of SLE patients and healthy controls. Table 2: the primer sequences of target genes used in qRT-PCR. Table 3: the amplification conditions of target genes using cDNA as a template in qRT-PCR. Table 4: the clinical and laboratory characteristics of SLE Jordanian patients. Table 5: the correlations between expression levels.
