# Supplementary material for: Correlations of Expression Levels of a Panel of Genes (IRF5, STAT4, TNFSF4, MECP2, and TLR7) and Cytokine Levels (IL-2, IL-6, IL-10, IL-12, IFN-γ, and TNF-α) with Systemic Lupus Erythematosus Outcomes in Jordanian Patients
Source: Biomed Res Int. 2019 Nov 29;2019:1703842. doi: 10.1155/2019/1703842 (PMC6907047; doi:10.1155/2019/1703842)
Supplement: Supplementary Materials — Table 1: the demographic data of SLE patients and healthy controls. Table 2: the primer sequences of target genes used in qRT-PCR. Table 3: the amplification conditions of target genes using cDNA as a template in qRT-PCR. Table 4: the clinical and laboratory characteristics of SLE Jordanian patients. Table 5: the correlations between expression levels. [file 1703842.f1.docx]

**Supplementary Table 1:** Demographic data of SLE patients and healthy controls

| **Parameter** | **Patients**  **(n=51)** | | **Controls (n=50)** |
| --- | --- | --- | --- |
|  | **High activity**  **(n=22)** | **Low activity**  **(n=29)** |  |
| **Age** | 31.6 ± 2.4 | 35.9±1.6 | 32.4 ± 1.1 |
| **Sex**  **Male**  **Female** | 1  21 | 2  27 | 3  47 |
| **Disease duration** | 9.0 ± 1.4 | 7.2±1.2 | - |

**Supplementary Table 2:** Primers sequences of target genes used in qRT-PCR.

| **Target gene** | **Forward primer(5`-3`)** | **Reverse primer (5`-3`)** | **References** |
| --- | --- | --- | --- |
| ***GAPDH*** | TGTTGCCATCAATGACCCCTTC | CTCCACGACGTACTCAGCGC | [67] |
| ***STAT4***  α-isoform  β-isoform | CATCTCAACAATCCGAAGTGATTCA  TGACCTTGTTATCTCTTTAAGCCGA | GTCAGAGTTTATCCTGTCATTCAGCAG  GTCAGAGTTTATCCTGTCATTCAGCAG | [37] |
| ***TNFSF4*** | GGTATCACATCGGTATCCTCGA | TGAGTTGTTCTGCACCTTCATG | [21] |

**Supplementary Table 3:** Amplification conditions of target genes using cDNA as template in qRT-PCR.

| **Target gene** | **Amplification conditions*** | | | | | |
| --- | --- | --- | --- | --- | --- | --- |
|  | **Pre-denaturation** | **Denaturation** | **Anealing** | **Elongation** | **Final extension** | **# cycles** |
| ***GAPDH*** | 95^o^C (5 min) | 95^o^C (10 sec) | 55^o^C (30 sec) | 72^o^C (30 sec) | 72^o^C (5 min) | 45 |
| ***STAT4***  α & β-isoforms | 95^o^C (5 min) | 95^o^C (15 sec) | 60^o^C (10 sec) | 72^o^C (30 sec) | 72^o^C (5 min) | 45 |
| ***TNFSF4*** | 95^o^C (5 min) | 95^o^C (15 sec) | 60^o^C (40 sec) | 60^o^C (40 sec) | 72^o^C (5 min) | 40 |

**Supplementary Table 4:** Clinical and laboratory characteristics of SLE Jordanian patients

| **Clinical and Laboratory characteristics(ACR criteria)** | **% Total patients**  **N=51** | **% High Activity**  **N=22** | **% Low Activity**  **N=29** |
| --- | --- | --- | --- |
| **Malar rash** | 45.1 | 15.7 | 29.4 |
| **Alopecia** | 21.6 | 3.9 | 17.6 |
| **Photosensitivity** | 27.5 | 3.9 | 23.5 |
| **Oral ulcers** | 45.1 | 21.6 | 23.5 |
| **Arthropathy** | 86.3 | 31.36 | 54.9 |
| **Serositis** | 15.7 | 9.8 | 5.9 |
| **Renal involvement** | 43 | 21.5 | 21.5 |
| **Neuropsychiatric** | 25.5 | 21.6 | 3.9 |
| **Hematological**  **Leucopenia**  **Anemia**  **Thrombocytopenia** | 9.8  31  17.7 | 3.9  15.7  5.9 | 5.9  15.7  11.8 |
| **Immunologic**  **Positive anti-dsDNA**  **Antiphospholipid antibodies**  **Anti-Sm antibodies**  **Positive ANA** | 26  44  20  96 | 9.8  16  10  43.1 | 15.7  28  10  52.9 |

**Supplementary Table 5**: Correlations between expression levels of SLE genes or cytokines with ANA, anti-dsDNA and SLEDAI score.

| **Gene** | | **R&P** | **ANA** | **Anti-dsDNA** | **SLEDAI score** | **Cytokine** |  | **ANA** | **Anti-dsDNA** | **SLEDAI score** |
| --- | --- | --- | --- | --- | --- | --- | --- | --- | --- | --- |
|  | ***IRF5*** | *R* | **-0.324*** | -0.236 | 0.155 | **TNF-α** | *R* | -0.065 | 0.143 | **-0.306^*^** |
|  |  | *P*-value | **0.022** | 0.100 | 0.275 |  | *P*-value | 0.654 | 0.320 | **0.029** |
| ***TLR7*** | | *R* | -0.195 | -0.078 | -0.042 | **IFN-γ** | *R* | 0.053 | -0.114 | -0.232 |
|  |  | *P*-value | 0.174 | 0.589 | 0.772 |  | *P*-value | 0.714 | 0.429 | 0.102 |
|  | ***MECP2 (α)*** | *R* | -0.003 | 0.137 | -0.037 | **IL-2** | *R* | -0.067 | -0.016 | 0.018 |
|  |  | *P*-value | 0.984 | 0.344 | 0.795 |  | *P*-value | 0.644 | 0.914 | 0.901 |
|  | ***MECP2(β)*** | *R* | -0.164 | -0.015 | 0.041 | **IL-12** | *R* | -0.233 | -0.094 | 0.285 |
|  |  | *P*-value | 0.254 | 0.915 | 0.777 |  | *P*-value | 0.104 | 0.516 | 0.065 |
|  | ***STAT4(α)*** | *R* | -0.003 | -0.037 | 0.041 | **IL-6** | *R* | **0.298^*^** | -0.194 | 0.182 |
|  |  | *P*-value | 0.983 | 0.801 | 0.776 |  | *P*-value | **0.036** | 0.178 | 0.202 |
|  | ***STAT4(β)*** | *R* | -0.005 | 0.040 | 0.015 | **IL-10** | *R* | 0.093 | -0.220 | **0.399^*^** |
|  |  | *P*-value | 0.974 | 0.785 | 0.919 |  | *P*-value | 0.522 | 0.124 | **0.015** |
| ***TNFSF4*** | | *R* | -0.006 | 0.042 | -0.069 | **IL10/IFN-γ** | *R* | - | - | **0.435^**^** |
|  |  | *P*-value | 0.969 | 0.770 | 0.631 |  | *P*-value | - | - | **0.001** |

*Correlation is significant at the 0.05 level (2-tailed), ** Correlation is significant at the 0.01 level (2-tailed), Spearman`s correlation coefficient.
